# Supplementary material for: Easy MPE: Extraction of Quality Microplot Images for UAV-Based High-Throughput Field Phenotyping
Source: Plant Phenomics. 2019 Nov 29;2019:2591849. doi: 10.34133/2019/2591849 (PMC7706339; doi:10.34133/2019/2591849)
Supplement: Supplementary Materials — Table S1: details about image acquisition of the trial fields. Table S2: Easy MPE program inputs for each dataset. [file 2591849.f1.docx]

Supplementary Materials

Table S1: Details about image acquisition of the trial fields

| Field | Camera model | Drone reference | Pix4D version | Number of drone images |
| --- | --- | --- | --- | --- |
| Dataset 1 | FC550 RAW DJIMFT 15 mm *f*/1.7 ASPH 15.0 4608 × 3456 (RGB) | DJI Inspire 1 | 4.2.25 | 156 |
| Dataset 2 |  |  | 4.1.24 | 210 |
| Dataset 3 | FC 6520 DJIMFT 15 mm *f*/1.7 ASPH 15.0 5280 × 3956 (RGB) | DJI Inspire 2 | 4.2.25 | 198 |
| Dataset 4 **(resized)** |  |  | 3.3.29 | 193 |
| Dataset 5 | FC 6310 8.8 5472 × 3648 (RGB) | DJI Inspire 1 | 4.2.26 | 121 |
| Dataset 6 |  |  | 4.2.26 | 121 |

Table S2: Easy MPE program inputs for each dataset

| Trial field | Type of image | Date of chosen image  (dd/mm/yyyy) | Noise (px) | Number of columns per microplot | Number of crop rows per microplot column | Column orientation |
| --- | --- | --- | --- | --- | --- | --- |
| Dataset 1 | RGB | 31/05/2017 | 200 | 1 | 2 | Vertical |
| Dataset 2 | RGB | 16/06/2017 | 100 | 1 | 2 | Vertical |
| Dataset 3 | RGB | 10/07/2017 | 1000 | 1 | 3 | Vertical |
| Dataset 4 | RGB | 10/07/2017 | 200 | 1 | 4 | Horizontal |
| Dataset 5 | Binary | 07/06/2018 | 700 | 1 | 2 | Vertical |
| Dataset 6 | Binary | 05/06/2018 | 500 | 1 | 2 | Vertical |
